# Where Are We Heading with Noninvasive Clinical Vascular Physiology? Why and How Should We Assess Endothelial Function?

**DOI:** 10.4061/2011/870132

**Published:** 2011-08-16

**Authors:** Elizabeth A. Ellins, Julian P. J. Halcox

**Affiliations:** Wales Heart Research Institute, Cardiff University, Heath Park, Cardiff CF14 4XN, UK

## Abstract

There are several invasive and noninvasive methods available to the clinical researcher for the assessment of endothelial function. The first investigations in humans involved invasive pharmacological vascular function testing, which have been used to gain a detailed understanding of the mechanisms involved in the pathogenesis of endothelial dysfunction and atherosclerosis as well as novel targets for intervention. Techniques for endothelial function testing have evolved over time from these invasive methods, which, by their nature, are restricted to small studies in the research laboratory, to more standardized noninvasive methods, which are suitable for use in large prospective cohort studies and clinical trials. This paper describes currently available methods for assessment of endothelial function and their potential application in cardiovascular research and clinical practice.

## 1. Introduction

The single-cell monolayer that lines the vascular system, the endothelium, is responsible for homeostasis of the blood vessels. It releases a number of factors that play a role in the regulation of vascular tone, the inhibition of platelet aggregation, and the adhesion of leukocyte. Disturbances in these processes cause endothelial activation and dysfunction, characterized by a decrease in the availability of the endothelium-derived vasodilator nitric oxide (NO) and the activation of vasoconstrictors such as endothelin-1 and angiotensin II. In combination with the expression of prothrombotic, proinflammatory, and adhesion molecules, this initiates an environment favourable to the development of atherosclerotic lesions. These lesions eventually develop into plaques, which can gradually progress to luminal obstruction and ischemia, or destabilize and rupture resulting in acute events such as myocardial infarction and stroke. Endothelial dysfunction is the earliest clinically identifiable event in the process of cardiovascular disease.

## 2. Rationale for Testing Endothelial Function

Furchgott and Zawadzki were the first to demonstrate that endothelial cells were required for smooth muscle relaxation to occur in response to acetylcholine administration in rabbit aorta [1]. Nitric oxide was later identified as the substance responsible for this relaxation [2]. Based on this and other works, Ludmer et al. were the first to show that acetylcholine caused vasoconstriction in atherosclerotic arteries and vasodilatation in healthy vessels in the human coronary circulation during cardiac catheterization [3]. Assessing changes in forearm blood flow in response to pharmacological probes allows a more detailed exploration of resistance vessel function, but these invasive studies are only appropriate for use in small study cohorts. In 1992, Celermajer et al. developed a noninvasive method for assessing endothelial function in the conduit arteries of the peripheral circulation [4]. This technique uses a period of forearm ischemia followed by reactive hyperemia to increase brachial arterial blood flow and, consequently, shear stress. This induces endothelial release of NO which mediates smooth muscle relaxation and vasodilatation of the brachial artery [5]. Since then other techniques have been developed that either employ reactive hyperemia or administration of drugs to stimulate the endothelium. Endothelial vasodilator function in the peripheral circulation has been shown to be related to coronary endothelial function and cardiovascular risk factors such as dyslipidemia, smoking, and diabetes and to be predictive of cardiovascular events [6–10].

## 3. Methods for Assessing Endothelial Function

Several invasive and noninvasive methods have been developed for the assessment of endothelial function. No single method is the perfect test, and a combination may be required for a comprehensive evaluation of vascular endothelial biology. 

### 3.1. Invasive Assessment of Endothelial Function

The original clinical investigations of endothelial vasomotor function assessed the coronary circulation. Changes in the epicardial and microvascular responses to endothelium-dependent pharmacological agents are measured during cardiac catheterization using quantitative coronary angiography and the Doppler flow-wire techniques. Preserved epicardial coronary endothelial function is characterized by vasodilatation in response to acetylcholine. Constriction of the vessel is indicative of the smooth muscle response to direct muscarinic receptor stimulation overwhelming the absent or depressed dilation that follows from reduced bioavailability of endothelial NO [11]. the use of this technique is realistically restricted to individuals in the more advanced stages of arterial disease as it should only be carried out in those with clinical indications for cardiac catheterization. However, coronary vascular function testing has provided important insights into the effects of atherosclerosis and its risk factors on coronary regulatory physiology and risk stratification as well as demonstrated the potential reversibility of endothelial dysfunction in response to treatments such as statins and ACE-inhibitors [12–14].

Forearm resistance vessels can be studied using venous occlusion plethysmography to assess changes in forearm blood flow (FBF) in response to pharmacological agents. This technique provides its own control by using the contralateral arm, permitting adjustments to be made for systemic influences that affect basal flow and blood pressure in the noninfused arm. The majority of studies measure percentage differences in FBF and vascular resistance between experimental and control arms following administration of endothelium-dependent and endothelium-independent agonists. Evaluation of the contribution of NO to vasomotor regulation can be made using eNOS antagonists such as L-NMMA. This technique can be used in healthy controls as well as patients, allowing the study of the endothelium from early in the disease process. It also allows other vasomotor pathways in addition to NO to be evaluated. However, it remains an invasive technique which limits its repeatability and also restricts its use to small studies. Also, the clinical relevance to atherosclerosis has been questioned, as microvascular pathophysiology may not necessarily reflect changes in the conduit arteries in which the plaques develop.

### 3.2. Noninvasive Methods of Assessment

**Figure 1:**
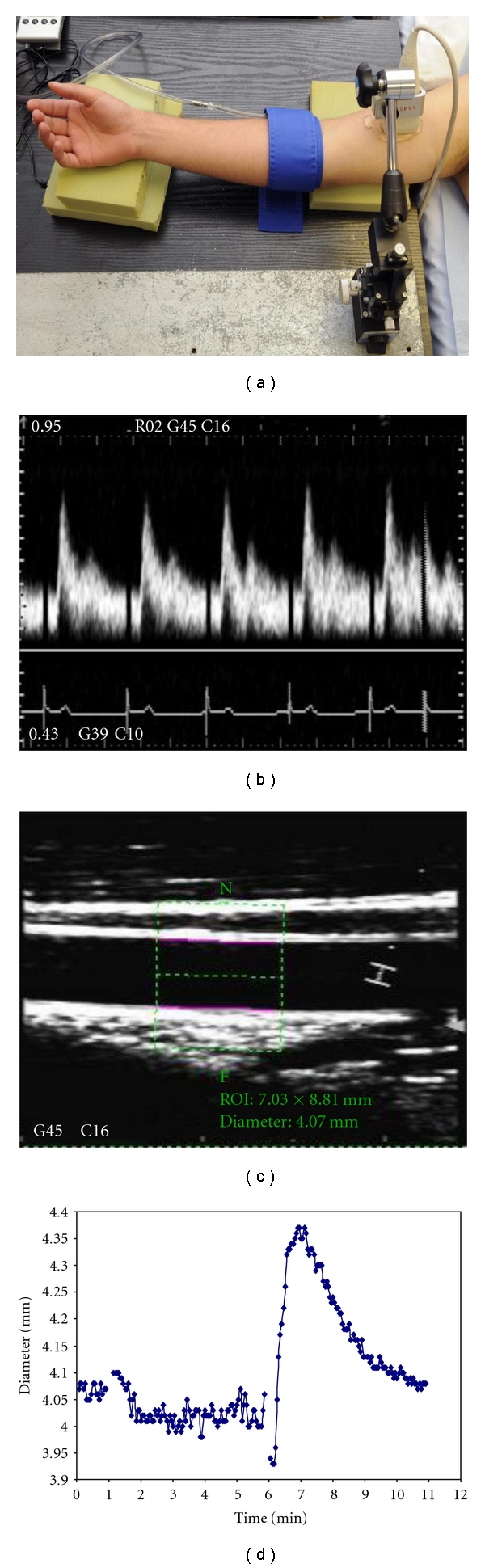
Setup and representative data for ultrasound evaluation of brachial FMD. (a) A sterotatic clamp with micrometer adjustment is used to hold the ultrasound probe. The occluding cuff is positioned just below the elbow. (b) Doppler trace showing flow velocity profile during early reactive hyperemia. (c) B-mode image of brachial artery demonstrating selection of region of interest and edge-detection analysis with Brachial analyzer software package. (d) Brachial artery diameter profile during FMD protocol as measured using brachial analyzer the software package (0-1 minute baseline, 1–6 minutes cuff up, 6–11 minutes response to reactive hyperemia (FMD)).

The current gold standard technique for noninvasive assessment of the endothelium is flow-mediated dilatation (FMD) using ultrasound. This method is based on the vasodilatation initiated by the reactive increase in blood flow in the brachial artery (or other conduit arteries) following a 5-minute period of forearm ischemia mediated by suprasystolic inflation of a blood pressure cuff on the arm. The resultant hyperemia following cuff release causes an increase in local shear stress in the vessel which stimulates the endothelium to generate and release NO, which activates guanylyl cyclase to produce cyclic GMP in vascular smooth muscle which causes relaxation and dilatation of the artery [5, 15]. The changes in blood flow and vessel diameter can be assessed by imaging the brachial artery and measuring blood flow with high-resolution 2D ultrasound and Doppler interrogation (Figure 1). It is also important to assess endothelium-independent vasodilatation for comparison, which reflects smooth muscle function, using the NO donor glyceryl trinitrate (GTN), usually administered sublingually. 

A technically demanding technique and initially expensive to set up is FMD which is normally used in the research laboratory where it has been demonstrated to have good reproducibility [16]. However, it can also be used with care in large epidemiological studies and has an expanding role in clinical trials [17–20]. Some differences in technique and controversies remain regarding the most robust methodology for FMD. Both cuff position and duration of the occlusion period can affect the reactive hyperemic stimulus. If the occlusion cuff is positioned above the ultrasound probe, making the whole arm ischemic including the measured arterial segment, a larger dilatation of the vessel is seen than when the cuff is placed on the forearm distal to the study segment [15, 21]. A cuff occlusion of 15 minutes also causes a larger hyperemic response than of a 5-minute period [22]. Thus, both proximal cuff positioning and longer occlusion periods may not specifically represent NO-mediated endothelium-dependent vascular function [15, 23]. In contrast, a more distal positioning of the cuff and using a 5-minute occlusion period have been demonstrated to induce NO-mediated vasodilatation, although there is still some debate over this matter [15, 22, 24–26]. Another area of current interest is how to interpret and adjust for the reactive hyperemic stimulus. Previously, only the maximal reactive hyperemic response was typically reported (if at all), but more recently it has been recommended that the impact of the change in shear stress, represented as shear rate, should be considered over the entire period of reactive hyperemia using the integral of shear rate over time (AUC) [27]. Efforts have been made to reduce the variability in the methodology, which can limit comparison of the results from different FMD studies [28–31].

**Figure 2:**
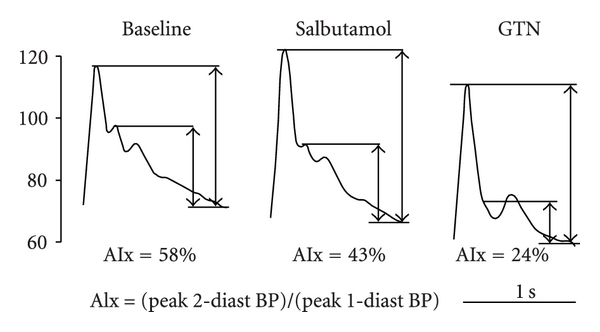
An example of the effect of salbutamol and glyceryl trinitrate (GTN) on the radial waveform in a single individual. The second systolic peak, obvious at baseline, is attenuated by salbutamol and almost completely abolished following GTN. Augmentation index (AIx), calculated as the ratio of the pulse pressure at the second systolic peak to that at the first systolic peak is used to quantify the changes in the waveform. BP = blood pressure. Printed with permission from [33].

Other methods of assessing peripheral vasomotor function involve the administration of agents such as the *β*2 adrenergic receptor agonist salbutamol, which can be administered via an inhaler or IV infusion and does not affect blood pressure at standard clinical doses [32–34]. Salbutamol stimulates the release of NO from the endothelium via vascular endothelial *β*2 receptor activation. This reduces arterial tone and stiffness, which can be measured in the peripheral waveform with either pulse wave analysis (PWA) by applanation tonometry at the radial artery or pulse contour analysis (PCA) with digital photoplethysmography (Figure 2). Assessment of the changes on the central aortic waveforms can also be measured by using a transfer function which has been validated only in adults. Despite these techniques' relative simplicity and low cost, there are practical concerns regarding reproducibility compared with FMD especially if using PCA and in children [16]. 

Additionally, little correlation was observed between FMD and results with these techniques, implicating distinct pathophysiological influences at different levels of the vasculature requiring further evaluation [16]. 

**Figure 3:**
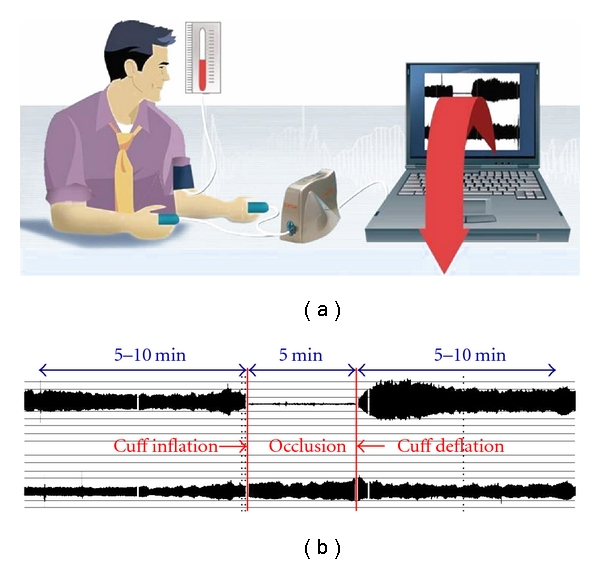
Diagram of the Endo-PAT set-up (a). (b) Representative traces from both the study and control arms showing the changes on the pulse amplitude after a 5-minute period of occlusion (printed with permission of Itamar Medical).

Pulse amplitude tonometry (PAT) is another technically straightforward technique, which uses the same stimulus as FMD. The Endo-PAT system uses a fingertip probe to measures changes in arterial pulsatile volume. Recordings are made simultaneously in the right and left index fingers so providing an internal control. As a measure of reactive hyperemia, similarly provoked by a 5-minute period of forearm ischemia, the RH-PAT index is calculated as the ratio of the average amplitude of the PAT signal over a 1-minute time interval starting 1 min after cuff deflation, divided by the average amplitude of the PAT baseline (Figure 3). RH-PAT index values from the study arm are normalized to the control arm. Reproducibility has been demonstrated to be similar to that of FMD, and mechanistically the vasodilation is mediated at least in part by NO [35, 36]. However, it is not entirely NO dependent, and there is likely to be an important interaction with the autonomic nervous system that may confound interpretation of the results from a specific endothelial perspective. Another limitation of the technique is the inability to take into account the impact of autonomic influences on endothelium-independent response to systemic GTN due to lack of a simultaneous unexposed control arm. 

Endothelial function can also be assessed using pulse wave velocity measurement. Arterial stiffness in the brachial artery reflects both arterial wall composition and smooth muscle tone. In response to a reactive hyperaemic stimulus, which increases shear stress and stimulates endothelial NO release, pulse wave velocity (PWV) slows due to the resultant drop in smooth muscle tone [37]. This simple technique, first developed by Naka et al. uses two cuffs, one placed at the wrist and one on the upper arm, to assess PWV in the arm; the reactive hyperaemic stimulus is induced by wrist cuff occlusion. An inverse relationship has been shown between PWV slowing and FMD using another method measuring carotid-radial PWV [38]. However these approaches still require further refinement and validation, but their relative simplicity is appealing for their potential application in larger-scale studies. 

Increases in skin blood flow in the distal forearm following local thermal hyperemia, reactive hyperaemia, or iontophoretic application of endothelial agonists can be measured using the laser Doppler flowmetry: allowing assessment of cutaneous microvascular endothelial function. However, local changes in blood flow are only in part due to NO, with prostanoids also mediating some of the response [39, 40]. Reproducibility for this technique has been shown to be similar to that of FMD [41].

In conclusion FMD is the current gold standard and most used method for noninvasive assessment of endothelial vasomotor function. There are other less expensive and technically simpler methods emerging, most notably,s Endo-PAT, but questions remain regarding which pathways are being assessed by these techniques as responses may not correlate with FMD or are not fully attenuated by NO inhibition. Furthermore, some of these techniques may also be less sensitive and reproducible than FMD, thus necessitating larger study cohorts.

## 4. Clinical Application of Endothelial Function Testing

How Do We Apply the Methodology in Clinical Studies, What Can It Show Us and How Do the Results Enhance Our Understanding of Vascular Biology, Atherosclerosis and CVD?

### 4.1. Role of Endothelial Function in Case-Control and Cohort Studies

Endothelial function testing was initially a research laboratory-based technique in relatively small case-control, cross-sectional, and interventional studies. However, with the improvement in technology allowing smaller and cheaper equipment and the development of semiautomated analysis techniques, endothelial function testing is now being included in large prospective cohort studies. Indeed, Donald et al. have recently shown that FMD can be measured with high reproducibility in a large pediatric cohort of just under 8,000 participants, the Avon Longitudinal Study of Parents and Children (ALSPAC) [20]. 

One of the first cohort studies to measure endothelial function was the Cardiovascular Health study which assessed FMD in 2792 72–98-year olds between 1997 and 1998. This study was primarily investigating factors related to the onset and course of coronary heart disease and stroke, but also evaluated the prognostic ability of FMD to predict cardiovascular events in older adults [9]. Another cohort study, the Firefighters and Their Endothelium (FATE), was intentionally set up to assess the relationship between endothelial function, emerging risk factors, and atherosclerotic vascular disease in 1600 middle-aged fire fighters [42]. Other established longitudinal studies have also incorporated endothelial function testing into their screening visits. Between them, these cohorts cover a wide range of ages and exposures from prenatal and early life (ALSPAC) through later childhood into adulthood (Young Finns' and Framingham's offspring studies) [20, 43, 44]. From a practical perspective, it may not always be appropriate or logistically possible to administer GTN to study endothelium-independent vasodilatation within these studies, which can limit the specificity of the observations.

### 4.2. Insights and Opportunities from Cohort Studies

Cardiovascular risk factors have long been associated with diminished endothelial function, confirmed more recently in the larger cohort studies [45, 46]. In those with impaired FMD, risk factor burden is associated with increased intima-media thickness, an important subclinical marker of atherosclerosis [47]. Not all associations, however, have been consistently replicated in cohort studies, for example, the relationship between attenuated endothelial function and elevated CRP found in small cross-sectional studies [48–51]. However, FMD is diminished in children with acute and recent minor infections [52].

The prospective nature of these studies allows researchers to look at the potential impact of earlier risk factor exposure on contemporary endothelial function. For example, childhood blood pressure was demonstrated to be predictive of endothelial dysfunction independent of presence and levels of other risk factors in adult life [53]. Associations found in cross-sectional analyses can also be followed up prospectively in the same cohort. For example, asymmetrical dimethylarginine (ADMA) was inversely correlated with FMD and subsequently found to predict FMD six years later [54, 55]. 

The prognostic ability of endothelial function testing can also be examined in prospective cohorts. Endothelium-dependent dilatation has been consistently shown to be significantly associated with progression of subclinical disease and incident clinical cardiovascular events in several studies [9, 56–58]. Although this association suggests a causally important influence on the pathogenesis of arterial disease, this alone is insufficient evidence to justify the use of endothelial function testing in routine clinical practice and data suggesting incremental predictive value, ideally with regard to improved ability of the results of such a test to reclassify the risk of an individual. Yeboah et al. demonstrated an overall 29% correct reclassification of subjects into low-, medium-, and high-risk groups with the addition of FMD to the Framingham Risk Score (FRS) in comparison to classification by FRS alone [59]. However, a more recent study found that only forearm resistance vessel endothelial function, and not FMD, was associated with 5-year risk of cardiovascular events [60]. It is important to note that in the latter study, the coefficient of variation for FMD was very high (29%) which may have limited the ability of the researchers to detect modest but important associations. This highlights very important practical concerns regarding the suitability of endothelial function testing for use in routine practice. The technical complexity of clinical endothelial function testing protocols together with the inherent physiological variability of endothelium-dependent vasodilatation under nonstandardized conditions means that such approaches are less practically suited for wider routine clinical use outside of the carefully controlled environment of a specialist vascular laboratory and currently remain predominantly in the research domain. 

FDA approval has been granted for the use of the EndoPAT device for noninvasive detection of patients with coronary endothelial dysfunction following a small study of 94 subjects with chest pain who underwent invasive coronary angiography and endothelial function testing [61]. In this cohort, an EndoPAT index threshold of 1.67 provided a sensitivity of 82% and a specificity of 77% for coronary endothelial dysfunction which may help guide the management of challenging patients with chest pain and angiographically unobstructed coronary arteries but cannot justify wider clinical application of this technology at present.

### 4.3. Role of Endothelial Function in Intervention Studies

Endothelial function has increasingly become an attractive endpoint for clinical studies. Indeed it is possible to detect changes in endothelial function relatively quickly in comparison to other longer-term endpoints such as changes in the carotid or coronary wall and occurrence of clinical cardiovascular events. It can also provide the opportunity to evaluate potential “pleiotropic” influences of drugs. For example, statins have been shown to improve endothelial function prior to and independent of their effects on the lipid profile [62, 63]. Other pharmacological interventions such as renin-angiotensin system antagonists, calcium channel blockers, antiplatelet drugs, and angina treatments (ranolazine) have also been shown to have beneficial effects on endothelial function in addition to their primary actions [19, 64–67]. However, results from such studies are not always consistent in effect and results may vary between agents and according to the vascular bed studied. For example, in the BANFF study, FMD only improved after treatment with quinapril and not enalapril or losartan, whilst in another study cilazapril did not significantly improve endothelial function in the peripheral resistance vessels [19, 68]. Thus, it is important to account for the potential heterogeneity of endothelial biology when interpreting the results of clinical interventions. These issues have been reviewed in greater depth elsewhere [18, 69]. Nonpharmacological interventions such as Omega 3 supplements and exercise have also been shown to enhance endothelial function, which may in part account for their prognostic benefits [70–73]. Study methodology is of course critical in the setting of interventional studies. The study protocol and equipment must be standardized, and observers should be trained to a high level of quality and reproducibility [74]. Having proven itself as an endpoint in intervention studies FMD is now being used as an endpoint in drug development trials (e.g., Dal-Vessel) in order to provide valuable biological insights at a relevant stage of the drug's clinical development [17]. Although a positive impact on endothelial function would not be an essential for further investment, an adverse outcome might prompt more cautious progression. Indeed, one might speculate that had a clinical endothelial function study been conducted at an earlier stage of the Torcetrapib development program, an adverse endothelial “signal” might have been detected at that point prompting a more cautious evaluation of the situation-given what we now know about the adverse impact of this agent on blood pressure and the renin-angiotensin system.

### 4.4. Endothelial “Stress Testing”: Opportunities to Gain Additional Insights into Vascular Pathophysiology

Recently, several experimental clinical models of acute endothelial dysfunction have been developed which allow exploration of endothelial behaviour in response to relevant, acute pathophysiological stimuli. These include ischemia reperfusion, inflammation, and acute psychophysiological stress. Application of these models either to explore drivers of disease progression and destabilization or to develop strategies to protect the cardiovascular system against these pathophysiological influences is still at an early stage but clearly has great potential [75–80]. 

## 5. Conclusion

The endothelium has an important influence on the development of atherosclerosis, and the assessment of its function affords a valuable insight into disease processes in the arterial wall. This has been exploited in a wide range of clinical situations and has more recently been used to good effect in large prospective cohort studies. It is important to emphasize that endothelial dysfunction, although important, is only a component of the pathophysiological process of atherogenesis. For example, inflammatory, proliferative, and thrombotic pathways acting independently of the endothelium also have important influences on plaque development, destabilization, and clinical sequelae. Unfortunately, the endothelium's inherent physiological sensitivity and complexity of some of the assessment methodologies make it a less suitable parameter to guide routine clinical practice, but it remains a core component of the clinical vascular research technique portfolio. Indeed, results from endothelial function testing provide insightful short- to medium- term outcome measures for detailed mechanistic vascular studies and early-phase clinical trials.
